# Restoration of gut microbiota with a specific synbiotic-containing infant formula in healthy Chinese infants born by cesarean section

**DOI:** 10.1038/s41430-025-01571-8

**Published:** 2025-02-06

**Authors:** Ying Wang, Harm Wopereis, Alexia Kakourou, Min Liu, Jieling Wu, Zailing Li, Lili Zhang, Meizhen Tan, June Su Yin Low, Mengjin Liu, Guus Roeselers, Jan Knol, Wei Cai

**Affiliations:** 1 Division of Pediatric Gastroenterology and Nutrition, Xinhua Hospital, School of Medicine, Shanghai Jiao Tong University, Shanghai, China; 2 Medical and Nutritional Science, Danone Research & Innovation, Utrecht, the Netherlands; 3 Obstetrics Department, Shanghai Public Health Clinical Center, Shanghai, China; 4 Department of Children Health Care, Guangdong Women and Children Hospital, Guangzhou, China; 5 Department of Pediatrics, Peking University Third Hospital, Beijing, China; 6 Department of Children Health Care, Wuxi Children’s Hospital, Wuxi, China; 7 Department of Children Health Care, Guangzhou Institute of Pediatrics, Guangzhou Women and Children’s Medical Center, Guangzhou Medical University, Guangzhou, China; 8 D-Lab, Danone Research & Innovation, Singapore, Singapore; 9 Danone Open Science Research Centre, Danone Research & Innovation, Shanghai, China

**Keywords:** Bacteria, Biomarkers

## Abstract

**Background:**

Birth by cesarean section (C-section) is associated with a delayed colonization of bifidobacteria and *Bacteroidota* species with potential negative health consequences. Previously, an infant formula with a synbiotic mixture of short-chain galacto-oligosaccharides and long-chain fructo-oligosaccharides (scGOS/lcFOS [9:1]) and *Bifidobacterium breve* M-16V was found to restore the timely colonization of bifidobacteria in C-section born infants. In this study, we investigated the effect of this synbiotic mixture on gut microbiota development in C-section and vaginally–born infants participating in a growth equivalence trial (NCT03520764).

**Methods:**

Healthy, fully formula-fed Chinese infants were randomized to receive a partially hydrolyzed whey-based protein formula with the synbiotic mixture (*n* = 112), or an intact protein formula with scGOS/lcFOS (prebiotic, *n* = 112). Breastfed infants served as reference group (*n* = 60). Gut microbiota profiling by 16S rRNA gene sequencing of stools collected at baseline, 17 weeks (end of intervention) and 12 months of age was complemented with analysis of stool pH, short-chain fatty acids, lactic acids, and secretory IgA concentrations.

**Results:**

Both the prebiotic and the synbiotic formula supported a dominant and diverse infant-type bifidobacterial community, but with increased abundance of *Bifidobacterium breve* in the synbiotic group. In C-section born infants (54.8% of randomized) the synbiotic but not the prebiotic, enhanced the bifidobacterial species diversity and showed restoration of *Parabacteroides* at 17 weeks, and *Bacteroides* spp. at 12 months closer to that of the vaginally–born infants.

**Conclusion:**

The synbiotic was confirmed to support the restoration of important gut colonizers in infants born by C-section with effects observed even beyond the intervention period.

## Introduction

Gut microbiome acquisition and development is significantly influenced by early life factors such as birth mode, antibiotics and breastfeeding [1]. Specifically, C-section birth leads to profound changes in gut microbiota development and is associated with an increased early-life risk of infections [2], adverse gastrointestinal symptoms [3], and later-life risk of developing non-communicable diseases including allergy [2], asthma [4] and obesity [5].

C-section delivery is characterized by a delayed colonization with *Bifidobacterium* species in the first months of life [6] and a reduced abundance of members of the phylum *Bacteroidota* (formerly *Bacteroidetes*) up to 1 year of age [7]. Often C-section delivery includes exposure of the newborn to maternally administered antibiotics which may contribute to the observed effects [8]. However, recent studies indicate that the compromised colonization of these taxa is mainly attributed to a disrupted vertical transfer from mother to infant [6]. Moreover, a postnatal catch-up of these taxa via the infants’ environment (horizontal transmission) is hampered due to their oxygen sensitivity and inability to form spores [9].

Species of *Bacteroidota* and *Bifidobacterium* have evolved genetic and enzymatic toolsets to utilize host-derived glycans, particularly human milk oligosaccharides (HMOs) [10]. This HMO degradation forms the foundation for the mutualistic symbiosis between humans and these keystone taxa [11]. After vaginal birth, these taxa rapidly expand in the gut of breastfed infants and contribute in maintaining an anaerobic gut environment [9]. The fermentation of HMOs by bifidobacteria lead to metabolites, such as acetate and lactate. These compounds effectively control the abundance of facultative anaerobic *Pseudomonadota* (formerly *Proteobacteria*), a phylum that includes several potential pathogens [12].

A recent clinical study by Chua et al. [13] demonstrated that a synbiotic mixture that combines the prebiotic short-chain galacto-oligosaccharides and long-chain fructo-oligosaccharides (scGOS/lcFOS, 9:1 ratio) with the probiotic *Bifdobacterium breve* M-16V can restore bifidobacteria colonization plus the gut physiology of C-section born close to that of vaginally-delivered infants [13]. In this study, we investigated effects of this specific synbiotic mixture added to a partially hydrolyzed whey-based protein formula (pHF) on gut microbiota development in a population of healthy Chinese infants participating in a previously published clinical trial [14]. This parent study confirmed that the synbiotic pHF was safe, well-tolerated and supported adequate growth [14]. Here we report 16S rRNA gene sequencing analyses performed on the fecal samples collected in this study. Additionally, the stool pH, short-chain fatty acids (SCFAs), lactic acids and secretory IgA (sIgA) concentrations were analyzed. More than half of the subjects in this study were born through C-section, which is in accordance with the high rates reported in China [15]. This prompted us to specifically investigate the effect of the intervention in relation to birth mode.

## Subjects and methods

### Stool samples

The stool samples analyzed were collected from healthy Chinese infants that participated in a double-blind, randomized controlled, two-arm parallel group, growth equivalence trial (ClinicalTrials.gov identifier NCT03520764). The study was conducted in compliance with the principles of the Declaration of Helsinki and all procedures were approved by Ethical Review Boards of participating hospitals. Before screening, written informed consent was obtained from parent(s)/legal guardians (parents) ≥18 y of age for each infant. Study design and subject inclusion was described in more detail before [14]. In short, this multicenter trial enrolled healthy, term-born, fully formula-fed infants (≤44 days of age) who received either the synbiotic pHF (Test, *n* = 112) or a commercially available intact protein formula (IF) containing the prebiotics only (Control, *n* = 112) until 17 weeks of age. Infants breastfed until at least 17 weeks of age formed the breastfed reference group (Breastfed, *n* = 60). The trial was powered to test equivalence in daily weight gain from baseline to 17 weeks of age as detailed previously [14]. The stool sample analysis described in this study was part of the exploratory outcomes of the trial.

Stools were collected at clinical visit 1 (baseline, before start of study product), visit 5 (17 weeks of age, end of intervention) and visit 8 (12 months of age, follow-up visit). Collection was done by the parents at home within 3 days prior to or on the day of the clinical visit using the stool collection kits provided at earlier visits. The samples were scooped from the diaper into 30-ml stool containers (Greiner 443102, Merck, Darmstadt, Germany) and immediately frozen at −12 °C or colder. Transport to the clinic was done using a cooler bag with frozen icepacks and stored in −80 °C freezers at the sites. Complete sample sets were shipped on dry ice to the laboratory of Applied Protein Technology Co. (Shanghai City, China) and stored at −80 °C until further sample processing and analysis. Once all samples were available, they were thawed once and aliquoted for the different types of analyses as described previously [16].

### Stool physiology and secretory IgA

The following stool physiology parameters were measured: pH, short-chain fatty acid (SCFA) concentrations (i.e., acetate, propionate, butyrate, isobutyrate, valerate, and isovalerate), D- and L-lactate concentrations, and secretory immunoglobulin A (sIgA). The analyses and quantification was performed by the laboratory of Applied Protein Technology Co. (Shanghai City, China) according to the methodologies as previously described in more detail for SCFAs [17], pH, lactic acids and SIgA [16], respectively.

### DNA extraction and 16S rRNA gene sequencing

DNA extraction from stool samples was performed with the QIAamp® Fast DNA Stool Mini Kit (Qiagen) according to the manufacturer’s protocol with additional bead-beating and an RNase incubation step as described previously [18]. DNA quality was checked using the NanoDrop™ spectrophotometer (Thermo Fisher Scientific, Waltham, Massachusetts, USA), whereas DNA concentrations were measured using the Qubit dsDNA BR Assay Kit (Thermo Fisher Scientific). DNA aliquots were stored at −80 °C and transported on dry ice in one batch to the BGI group (Shenzhen, China) for 16S rRNA gene sequencing analysis. Hypervariable V3–V4 regions of the 16S rRNA gene were sequenced on the MiSeq™ platform (Illumina, San Diego, California, USA) as described previously [19].

### Bioinformatics

The resulting sequencing reads were analyzed following the workflow detailed in the Supplementary Methods. In summary, the read pairs were demultiplexed and trimmed before being merged. High quality merged reads were dereplicated and counted, after which filtering was applied to remove reads with less than two reads over all samples, chimeras, and reads including PhiX or adapter sequences. Taxonomic assignment of sequence reads was performed using the RDP classifier [20] against the SILVA database [21] up to the genus level. Unsupervised oligotyping of *Bifidobacterium* assigned sequences up to the species level was performed using the Minimum Entropy Decomposition (MED) algorithm [22]. Alpha-diversity and beta-diversity metrics were calculated using the phyloseq v.1.38.0 [23] and vegan v.2.6-2 [24] packages in R v.4.1.0 [25].

### Statistics

The stool physiology, sIgA and alpha-diversity parameters were analyzed using a linear mixed-effects model for repeated measures (MMRM) including intervention, visit and infant age at baseline as fixed main effects, intervention by visit as a fixed interaction effect and subject as a random effect. A modifier analysis was conducted by including birth mode as main fixed effect plus its interaction with intervention, and intervention by visit. Stool parameters that could not be analyzed adequately with a MMRM (on the original or log-scale) were compared by the Wilcoxon rank sum test.

Principal coordinate analysis (PCoA) and distance-based redundancy analysis (db-RDA), using Bray–Curtis metrics were applied to compare the relative taxonomic compositions (beta-diversity) between samples per visit. Permutational multivariate analysis of variance (PERMANOVA) followed by post-hoc comparisons with Holm’s step-down correction for multiple testing was applied to compare the intervention and breastfed reference group centroids. Time-dependent microbial community responses and the interaction of these groups with birth mode were assessed with a principal response curves (PRC) analysis using the weighted Aitchison log-ratio method with statistical significance assessment by Monte Carlo permutation testing [26, 27].

Differential taxon abundances for the intervention at the different visits was analyzed using the multi-part statistical test as described previously [28]. Responder taxa identified with the PRC analysis were analyzed using generalized linear mixed-effects models for repeated measures (GLMM) using a negative binomial (NB) or a zero inflated negative binomial (ZINB) regression model depending on the magnitude of zero counts for each taxon. Model formulation was similar to that of the MMRM modifier analysis with the logarithm of the taxon count as outcome and the logarithm of sequencing depth as offset to weight for the differences in sampling effort.

All statistical analyses were conducted according to a predefined statistical analysis plan finalized before data unblinding and were performed using SAS® (Life Science Analytics Framework version 9.4, SAS Institute, Cary, NC, USA) except for the beta-diversity analysis and (zero-inflated) negative binomial models, which were analyzed with Canoco software v.5.15 for multivariate exploration and visualization [29] and R v.4.1.0 using the following packages: phyloseq v.1.38.0 [23], vegan v.2.6-2 [24], tidyverse v.1.3.1 [30], glmmTMB v.1.0.2.1 [31], emmeans v.1.6.0 [32] and DHARMa v.0.4.1 [33].

## Results

### Analysis population

Stools collected from all subjects randomized (ASR) and the breastfed reference were analyzed if at least one stool sample per subject was available and collected within the predefined age-categories of the visits, i.e., baseline (1–44 days), visit 5 (17 weeks ± 14 days), and visit 8 (12 months ± 28 days). The resulting analysis population consisted of 221 out of 224 subjects of the ASR population (Test *n* = 112, Control *n* = 109), and 58 out of 60 subjects of the breastfed reference with at least one non-missing stool parameter. Most of these subjects had stool samples collected at all three visits, with the majority of those randomized being fully formula-fed from birth onwards (53.6% in the Test group and 52.3% in the Control group) prior to the start of study product. Also, the pre-study duration of any breastfeeding was similar between Test and Control, and on average, the full study product was introduced at 21 (SD = 14.8) days of age (Table 1).

**Table 1:** Subject and maternal characteristics across study groups.

	Statistic	Test	Control	Total	Breastfed
Total analysis population	*N*	112	109	221	58
Analysis population at baseline	*n*	112	108	220	58
Analysis population at 17 weeks	*n*	90	95	185	52
Analysis population at 12 months	*n*	89	94	183	53
Number of stools collected across the 3 visits					
Subjects with 1 stool	*n* (%)	18 (16.1%)	13 (11.9%)	31 (14%)	5 (8.6%)
Subjects with 2 stools	*n* (%)	9 (8%)	4 (3.7%)	13 (5.9%)	1 (1.7%)
Subjects with 3 stools	*n* (%)	85 (75.9%)	92 (84.4%)	177 (80.1%)	52 (89.7%)
Age at time of stool sampling					
Baseline – 1 to 44 days of age	Mean (SD)	16.3 (13.3)	19.9 (14.8)	18.0 (14.1)	20.0 (8.1)
17 weeks – 106 to 133 days of age	Mean (SD)	119.8 (3.8)	119.2 (3.9)	119.5 (3.8)	119.3 (3.3)
12 months – 337 to 393 days of age	Mean (SD)	365.8 (7.9)	367.0 (7.8)	366.4 (7.8)	369.8 (7.7)
Sex (Female)	*n* (%)	60 (53.6%)	55 (50.5%)	115 (52.0%)	35 (60.3%)
Birth mode (C-section)	*n* (%)	71 (63.4%)	50 (45.9%)	121 (54.8%)	26 (44.8%)
Maternal IAP (yes)	*n* (%)	61 (54.5%)	61 (56.0%)	122 (55.2%)	18 (31.0%)
CS and IAP	*n* (%)	58 (51.8%)	46 (42.2%)	104 (47.1%)	9 (15.5%)
VD and IAP	*n* (%)	3 (2.7%)	15 (13.8%)	18 (8.1%)	9 (15.5%)
Antibiotics within 30 days before sample date					
Baseline (yes)	*n* (%)	3 (2.7%)	1 (0.9%)	4 (1.8%)	1 (1.7%)
17 weeks (yes)	*n* (%)	5 (5.6%)	4 (4.2%)	9 (4.9%)	0 (0.0%)
12 months (yes)	*n* (%)	5 (5.6%)	5 (5.3%)	10 (5.5%)	7 (13.2%)
Siblings in study (yes)	*n* (%)	22 (19.6%)	6 (5.5%)	28 (12.7%)	2 (3.4%)
Non-twin siblings	*n* (%)	2 (1.8%)	0 (0%)	2 (0.9%)	0 (0%)
Twin siblings	*n* (%)	20 (17.9%)	6 (5.5%)	26 (11.8%)	2 (3.4%)
Allergy in the family (yes)	*n* (%)	28 (25%)	40 (36.7%)	68 (30.8%)	23 (39.7%)
Subject is fully FF from birth (yes)	*n* (%)	60 (53.6%)	57 (52.3%)	117 (52.9%)	0 (0%)
Duration any BF (days)	Mean (SD)	32.3 (48.2)	39.2 (72.3)	35.8 (61.2)	321 (94.8)
Start of SP before baseline visit (yes)	*n* (%)	1 (0.9%)	1 (0.9%)	2 (0.9%)	0 (0.0%)
Age at start any SP (days)	Mean (SD)	17.1 (13.0)	20.5 (14.7)	18.8 (14.0)	n/a
Age at start full SP (days)	Mean (SD)	19.4 (13.9)	22.5 (15.6)	21.0 (14.8)	n/a
Duration full SP (days)	Mean (SD)	126 (41.4)	122 (43.9)	124 (42.6)	n/a
Age at start complementary feeding (weaning)	Mean (SD)	172 (36.3)	175 (36.9)	173 (36.5)	184 (26.7)

The column ‘Total’ represent the combination of randomized groups (Test + Control). *N* = total number of subjects in the analysis population, defined as subjects from the All Subjects Randomized (ASR) and breastfed populations from whom at least one stool sample was available and collected within visit window and having at least one stool parameter with a non-missing result. Data are presented as mean (SD) or number (*n*) with percentages (%) relative to total number of subjects in the analysis population (*N*) or relative to the number of subjects in the analysis population at the respective visits (*n*).

*IAP* intrapartum antibiotic prophylaxis, *CS* C-section delivery, *VD* vaginal delivery, *FF* formula feeding, *BF* breastfeeding, *SP* study product, *‘n/a’* not applicable.

Subject characteristics were generally well balanced with some exceptions as previously reported [14], i.e. Test was slightly younger at baseline (t-test, *p* = 0.056), had a higher percentage siblings (Fisher exact test, *p* = 0.002) and C-section deliveries (Fisher exact test, *p* = 0.010) compared to Control [14]. About half of the randomized subjects were born through C-section (*n* = 121, 54.8%), of whom the majority (*n* = 104, 86%) were exposed to intrapartum antibiotics, administered prophylactically to the mother’s, in accordance with common hospital guidelines for C-section deliveries [8].

### Intervention effects in the total analysis population

#### Gut microbiota composition and diversity

DNA was successfully extracted and sequenced from 669 stool samples from 273 subjects with a median sequencing depth of 50,600 reads per sample (range: 12,049–65,969) (Supplementary Table 1). Taxonomic profiling resulted in 100 bacterial genera (Supplementary Table 2) with on average 17 genera per sample at baseline (SD = 5.07), 22 at 17 weeks (SD = 5.09) and 36 at 12 months (SD = 7.36). The microbiota composition across all groups and visits was characterized by dominant levels of *Actinomycetota* (formerly *Actinobacteria*, mostly *Bifidobacterium* genus), followed by *Pseudomonadata* (formerly *Proteobacteria*), *Bacillota* (formerly *Firmicutes*), and *Bacteroidota* (formerly *Bacteroidetes*) (Supplementary Fig. 1a), which is in accordance with patterns reported across global infant populations [7].

**Fig. 1:**
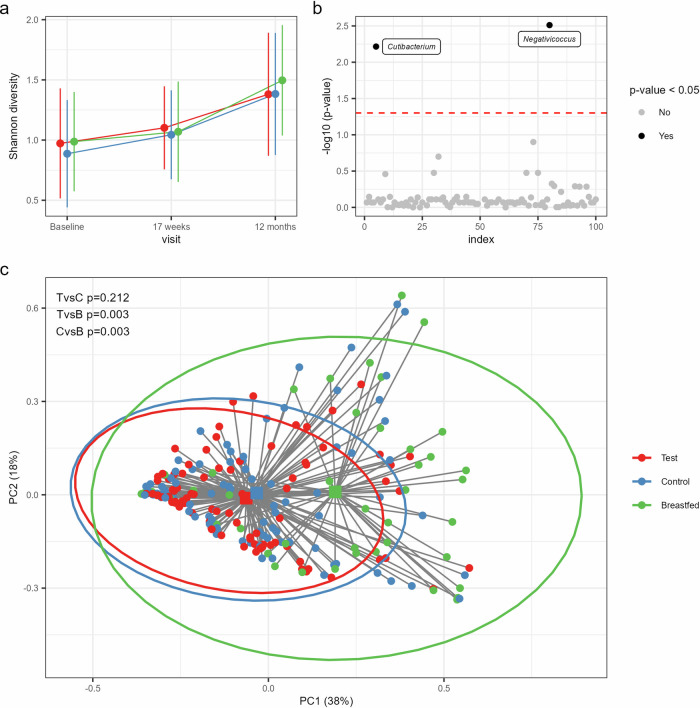
Similar development of gut microbiota comparing infants receiving the synbiotic with the prebiotic formula. **a** Line plots showing bacterial diversity in the study groups across the visits based on the Shannon index (mean with SD): Test (red), Control (blue) and Breastfed reference group (green). **b** Taxa with significantly different relative abundances between Test and Control at 17 weeks of age based on the multi-part statistical test with correction for multiple testing by controlling the positive false discovery rate at 5% (red dashed line). Significant taxa are shown by black dots with taxon assignments and non-significant taxa by gray dots. **c** Comparison of gut microbiota composition at 17 weeks based on a Principal Coordinate Analysis (PCoA) with Bray–Curtis sample distances. The two primary axes (PC1 and PC2) together account for 56% of the total microbial variance. Individual samples are shown as dots with their distances (gray lines) to the group centroids (squares) and the 95% confidence interval ellipses per study group. The *p*-values represent the results of the post-hoc comparisons from the PERMANOVA test on the group centroids with Holm’s step-down adjustment to correct for multiple testing at a 5% threshold. TvsC Test *vs*. Control, TvsB Test *vs*. Breastfed, CvsB Control *vs*. Breastfed.

No statistically significant difference in alpha-diversity was observed between the intervention groups (Fig. 1a). In general, alpha-diversity in both formula groups showed an increment across visits comparable to the breastfed reference group. Principal coordinate analysis (PCoA) based on Bray–Curtis distances at genus level showed significant differences between the group centroids of Test and Control at baseline (PERMANOVA, *p* = 0.039, Supplementary Fig. 1b), but not 17 weeks (Fig. 1c) nor at 12 months of age (Supplementary Fig. 1c). At 17 weeks, both formula groups showed similar distances to the breastfed reference, which was confirmed by pairwise comparisons of their group centroids (*p* = 0.003 for both comparisons). No significant differences with breastfed were observed at baseline or 12 months.

Since Test and Control showed a significant compositional difference at baseline, additional group comparisons at 17 weeks and 12 months were performed by applying distance-based redundancy analyses (db-RDA) with correction for age at baseline and birth mode to account for their imbalance between Test and Control. Indeed, both factors explained a significant part of the variation in taxonomic composition at baseline (*p* = 0.001 for both covariates), and birth mode was still significant at 17 weeks (*p* = 0.001) and 12 months (*p* = 0.01). After partialling out these confounders (including the residual group differences at baseline) in a db-RDA at 17 weeks and 12 months confirmed that Test and Control were compositionally similar at these visits.

Differential abundance analysis comparing Test with Control at each visit using the multi-part statistical test revealed significantly different abundances at 17 weeks of two low-abundant bacterial genera, i.e., *Cutibacterium* and *Negativicoccus* (*p* = 0.006 and *p* = 0.003, respectively). Both taxa showed increased presence (*Cutibacterium*: 38% *vs*. 20% and *Negativicoccus*: 45% *vs*. 20%) and relative abundances (mean with (SD) for *Cutibacterium*: 0.05 (0.15)% *vs*. 3.9E−03 (0.02)% and *Negativicoccus*: 0.16 (0.34)% *vs*. 0.03 (0.17)%) in Test *vs*. Control (Fig. 1b).

#### Bifidobacterium species diversity and composition

Unsupervised oligotyping of the bifidobacterial sequences was applied to discriminate *Bifidobacterium* species [22]. In total, 59 bifidobacterial oligotypes were identified (Supplementary Table 3) with an average of 11 oligotypes per sample at baseline (SD = 9.36), 19 at 17 weeks (SD = 11.8) and 23 at 12 months of age (SD = 13.9).

**Fig. 2:**
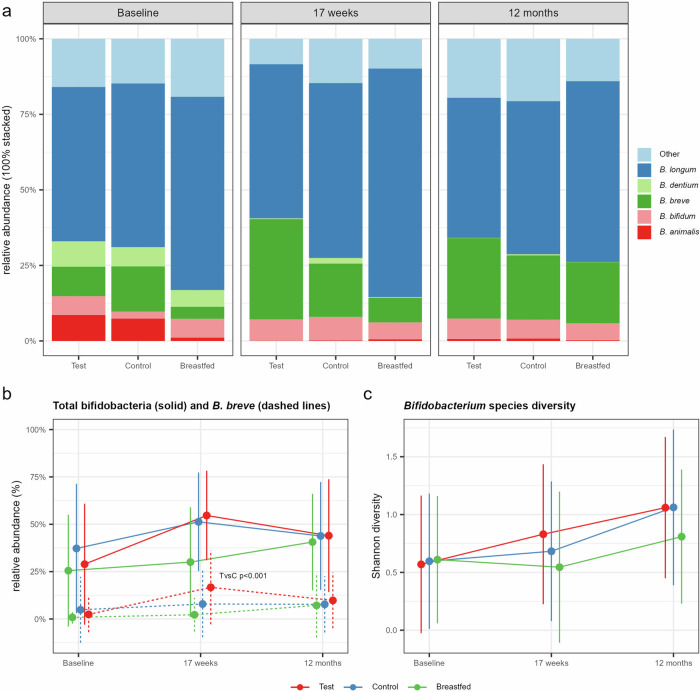
*Bifidobacterium* community identified by oligotyping of the bifidobacterial 16S rRNA sequences. **a** Stacked barplot summarizing the bifidobacterial species composition relative to all bifidobacteria by study group and visit. Bifidobacterial oligotypes with the same taxonomic assignments were aggregated at the species level. Oligotypes without taxonomic assignments are aggregated to “Other”. **b** Line plots for Test (red), Control (blue) and the Breastfed reference (green) group showing the relative abundance (mean with SD) of *Bifidobacterium* spp. (solid lines) and *B. breve* (dashed lines) relative to all taxa. Significant difference was observed at 17 weeks for *B. breve* comparing Test with Control (TvsC) using the multi-part statistical test. **c** Line plots for *Bifidobacterium* species diversity based on the Shannon index (mean with SD) calculated on identified bifidobacterial oligotypes.

In total, 40 of the low abundant oligotypes could not be reliably assigned taxonomically to species level. The majority of the remaining oligotypes were assigned to *B. longum*, followed by *B. breve* and *B. bifidum* (Fig. 2a). The total relative abundance of bifidobacteria increased from baseline in both Test and Control and were equally abundant at 17 weeks, but showed a significant increase of *B. breve* in the synbiotic compared to the prebiotic formula, which likely reflects the delivery of the probiotic strain (*p* < 0.001, Fig. 2b). The bifidobacterial species diversity was highest in both formula groups at 17 weeks and 12 months and showed a non-significant increase in Test compared to Control at 17 weeks (Shannon diversity, *p* = 0.057, Fig. 2c). The modifier analysis that included birth mode into the model revealed that the increase of bifidobacterial species diversity at 17 weeks was mainly attributed to a significant increase in the C-section born subgroup receiving the synbiotic formula (Test *vs*. Control, *p* = 0.0256) with levels closer to that of the vaginally delivered infants receiving either the synbiotic or the prebiotic formula (Supplementary Fig. 2). Overall, the breastfed reference was associated with a lower bifidobacterial species diversity and a community more dominated by *B. longum*, which reflects the central role of this species, specifically its subspecies *B. infantis*, in HMO degradation [34].

#### Stool physiology and secretory IgA

Stool physiology and sIgA were mostly similar between Test and Control, except for the concentrations of acetate and L-lactate, which were significantly lower (*p* = 0.0067 and *p* = 0.0088) (Supplementary Table 4), and the concentrations of branched-chain short chain fatty acids (isobutyric and isovaleric acid), which were significantly higher (*p* = 0.015 and *p* = 0.006, respectively) in Test compared to Control at 17 weeks. These observations were also confirmed with additional correction of the linear models for the covariates age at baseline and birth mode.

### Intervention effects by birth mode

#### Altered development of gut microbiota in C-section born infants

**Fig. 3:**
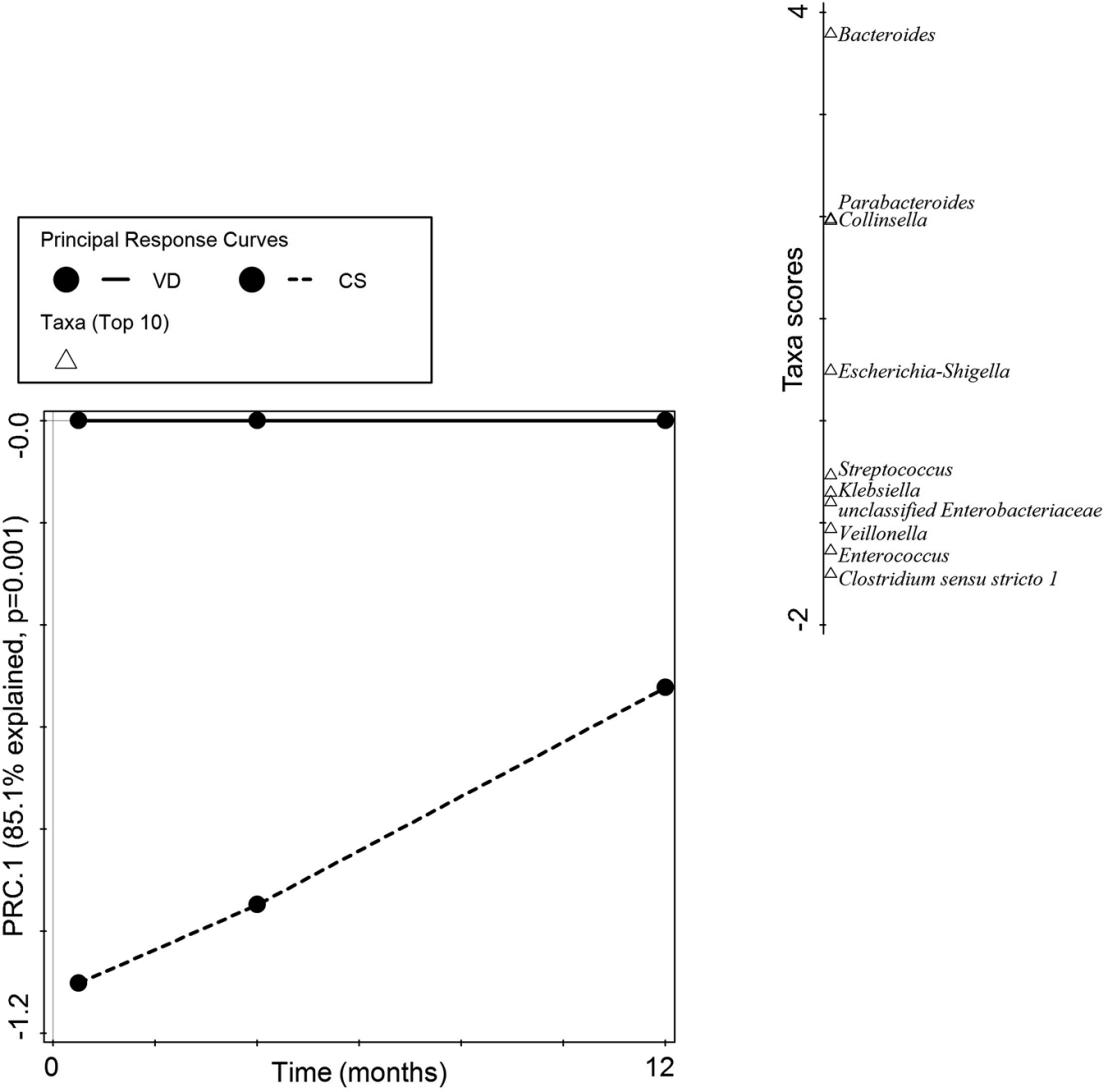
Altered development of gut microbiota in C-section born infants. First component (PRC.1) of the Principal Response Curves (PRC) analysis summarizing the response of the microbial community in C-section delivered (CS, dashed line) compared to vaginal delivered infants (VD, solid reference line) across time (horizontal axis). The interaction of birth mode with visit was significant (*p* = 0.001) and explained 85.1% percent of the total variation in genus composition on the first component. The top 10 taxa with the highest contribution to the first component are shown on the separate vertical axis (Taxa scores). Taxa with a positive score are decreased across time, and taxa with a negative score are increased across time in CS compared to VD infants, respectively.

The relatively high rate of C-section born infants in this analysis population prompted us to further investigate the effects of birth mode on gut microbiota development in ASR and the breastfed reference. Principal response curves (PRC) analysis showed a significant interaction of birth mode on the community response across visits (*p* = 0.001, Fig. 3). Specifically, C-section delivery was associated with a reduced relative abundance up to 1 year of age of two genera of the phylum *Bacteroidota* (*Bacteroides* and *Parabacteroides*) and *Collinsella*, while increased relative abundances were observed for *Clostridium sensu stricto* 1, *Enterococcus*, *Veillonella*, *Streptococcus* and members of the phylum *Pseudomonadota* (*Klebsiella*, and unclassified *Enterobacteriaceae*, respectively).

#### Restoration of gut microbiota with synbiotics

**Fig. 4:**
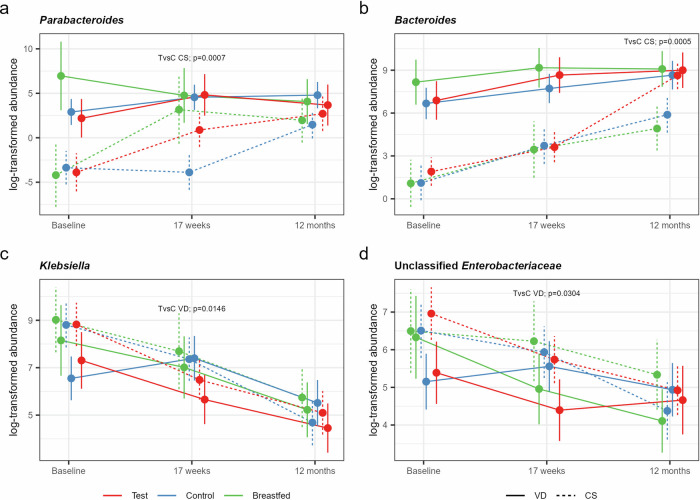
Differential responses of taxa comparing the synbiotic with the prebiotic formula by birth mode. Mean log-transformed abundance estimates (with 95% CL) for **a** Parabacteroides*, **b** *Bacteroides*, **c** *Klebsiella*, and **d** unclassified *Enterobacteriaceae* for the Test (red), Control (blue) and the Breastfed reference group (green) born by C-section (CS, dashed lines) or vaginally (VD, solid lines) with statistical assessment of significant treatment differences based on a (zero-inflated*) negative binomial (NB) mixed-effect model for repeated measures.

Next, we investigated the effects of the study formulas on these specific taxa within the C-section and vaginally delivered subgroups. The synbiotic formula was observed to restore the levels of the two *Bacteroidota* genera in C-section born closer to that of the vaginally-delivered infants (formula-fed and breastfed) with significantly increased relative abundance of *Parabacteroides* at 17 weeks (*p* = 0.0007, Fig. 4a) and *Bacteroides* at 12 months (*p* = 0.0005, Fig. 4b) compared to C-section born infants receiving Control formula. C-section delivered breastfed infants also showed restoration of *Parabacteroides*, but not *Bacteroides*. No significant effects were observed for the other taxa identified to be affected by birth mode. However, comparing Test with Control within the vaginally born subgroup showed significantly decreased relative abundances of *Klebsiella* (*p* = 0.0146, Fig. 4c) and unclassified *Enterobacteriaceae* (*p* = 0.0304, Fig. 4d) in the synbiotic group.

## Discussion

In this prospective, randomized, double-blind, controlled trial we investigated microbiota composition and activity of infants receiving a pHF supplemented with the prebiotic mixture scGOS/lcFOS (9:1) and the probiotic strain *B. breve* M-16V compared with infants receiving an intact protein formula solely containing the prebiotic mixture. Wang et al. [14] showed that the synbiotic pHF supported adequate growth, was well tolerated, and suitable for use. Here we show that the effect of both formulas are in line with previous studies investigating the effects of the specific prebiotic [35] and synbiotic mixture [13, 36, 37] with increased levels of bifidobacteria and its metabolites L-lactate and acetate resulting in a mildly acidic gut pH, akin to breastfed infants [35]. Interestingly, the synbiotic compared to the prebiotic formula showed a more diverse bifidobacterial community with an increased abundance of *Bifidobacterium breve* indicating the successful delivery of the probiotic strain without the competitive exclusion of endogenous infant-type bifidobacterial species, such as *B. longum* and *B. bifidum*. The synbiotic-associated increase of bifidobacterial diversity was mainly attributed to an enrichment from baseline to 17 weeks in the C-section subgroup, bringing it closer to levels observed in vaginally-delivered infants receiving either the prebiotic or synbiotic formula. Moreover, the synbiotic also restored members of the phylum *Bacteroidota* in the C-section subgroup. Specifically, (i) *Parabacteroides* at 17 weeks, and even beyond the intervention period (ii) *Bacteroides* at 12 months, reaching levels similar to those observed in both formula-fed and breastfed vaginally-delivered infants.

In the total analysis population, some minor differences for the synbiotic *vs*. the prebiotic formula were observed, i.e. an increased abundance of *Cutibacterium* spp. and *Negativicoccus* spp., along with increased branched-chain SCFAs, which may be attributed to differences in protein composition (whey vs. whey-casein and partial vs. intact protein). Branched-chain SCFAs are derived from branched-chain amino acids and therefore provide an indicator of protein fermentation activity [38], and both bacterial taxa have been correlated with protein-degrading activity [39, 40]. Although increased colonic protein fermentation is generally considered unfavorable [41], the difference in branched-chain SCFAs did not correlate with more potential pathogens. Instead, in the vaginally-born subgroup, there was a reduction of *Pseudomonadata* spp., including *Klebsiella*, supporting reports of synbiotic-enhanced colonization resistance against potential pathogens [37].

The microbiota responses following C-section delivery in this study align with previous findings on birth mode’s impact on infant gut microbiota development. Our results highlight compromised maternal transfer of key taxa, including *Bifidobacterium* and *Bacteroidota* [6, 42–45]. Chua et al. [13] previously showed that the synbiotic in a 16-week postnatal intervention recovered bifidobacterial colonization in C-section born infants from the first days of life, and associated with a reduction of skin disorders. However, no recovery was observed for *Bacteroidota* [36]. In this study, we did observe a recovery of *Bacteroidota* from 17 weeks onwards, suggesting that the synbiotic formulation can still partially correct the lack of *Bacteroidota* at a later stage of gut microbiota development.

The current study had limitations: it was not powered to evaluate gut microbiota effects of the synbiotic formula upon C-section delivery. However, the observations suggest specific effects that warrant further investigation. For effective restoration in C-section infants, the intervention ideally should start right after birth and more frequent stool sampling would give better insights into the microbiota trajectories. Furthermore, studies with longer-term follow-up are needed to confirm the potential health benefits associated with the timely restoration of keystone taxa with this specific synbiotic.

Our study confirmed that both prebiotic and synbiotic formulas promote healthy gut microbiota development. Specifically, the synbiotic formula led to a dominant and diverse infant-type bifidobacterial community, with increased *Bifidobacterium breve* abundance. Moreover, the synbiotic restored *Bacteroidota* abundance in C-section-born infants, approaching levels observed in vaginally born infants.

## Supplementary information

Supplementary Methods

Supplementary Figures

Supplementary Table 1

Supplementary Table 2

Supplementary Table 3

Supplementary Table 4

## Data Availability

The 16S rRNA-gene sequencing data of this study have been deposited in the European Nucleotide Archive (ENA) at EMBL-EBI under accession number PRJEB61020. The clinical study data supporting the findings of this study are available on request from the corresponding author; understanding that there could be reasonable caveats for such requests. Researchers that meet the criteria for access to confidential clinical study data must be compliant with the Danone Research & Innovation Clinical Trial Dataset Sharing policy and the Regulation on Human Genetic Resources issued by the Human Genetic Resources Administration of China.
