# Supplementary material for: Restoration of gut microbiota with a specific synbiotic-containing infant formula in healthy Chinese infants born by cesarean section
Source: Eur J Clin Nutr. 2025 Feb 6;79(6):567–75. doi: 10.1038/s41430-025-01571-8 (PMC12151850; doi:10.1038/s41430-025-01571-8)
Supplement: Supplementary file 1 — Supplementary Methods [file 41430_2025_1571_MOESM1_ESM.docx]

# Supplementary Methods

## 16S rRNA gene sequencing and bioinformatics

From the fecal DNA extracts, the hypervariable V3–V4 regions of the 16S rRNA gene were amplified and sequenced following the 16S Metagenomic Sequencing Library Preparation protocol (Illumina, Part # 15044223 Rev. B) with some modifications. In brief, the V3-V4 region of the 16S rRNA gene was PCR-amplified using the universal primers S-D-Bact-0337-a-S-20 (forward primer 5’-ACTCCTACGGGAGGCAGCAG-3’) and S-D-Bact-0787-b-A-20 (reverse primer 5’-GGACTACHVGGGTWTCTAAT-3’) (1). The amplicons were sequenced on the MiSeq™ platform (Illumina, San Diego, California, USA) in the 2 x 300 bp paired-end mode.

The resulting sequencing reads were demultiplexed and subsequently trimmed using cutadapt v.2.6 (2) by removing the primers and adapters and truncating reads with a mean phred q-score lower than 20 over a window of 30 bases. Trimmed reads with a length less than 75% of the original length, as well as low-complexity reads (>10 identical consecutive bases) and reads with ambiguous bases were removed. Merging of the cleaned paired-end reads was performed using PEAR v.0.9.8 (3) and the resulting consensus sequences were retained for reads with a minimum length of 300 and a q-score greater than 25 over a window of 15 bases. These merged reads were dereplicated into unique sequences (unique.seqs) and counted using Mothur v.1.41.1 (4). Unique sequences with a low abundance (counted < 2 times across all samples) were discarded. Chimeric sequences were removed using VSEARCH v.2.4.1 (5) with the RDP gold database (6) as reference. Sequences which contained PhiX or adapters as defined in Deblur (7) as well as sequences with a low relative abundance up to 0.0005% were removed. Taxonomic assignment was performed using the RDP classifier (8) against the SILVA138 database (9) up to the genus level. Unsupervised oligotyping of *Bifidobacterium* assigned sequences to discriminate bifidobacterial species was performed using the Minimum Entropy Decomposition (MED) algorithm (10) with taxonomic assignment using the RDP classifier (8) against the SILVA132 database (9) up to the species level. Bray-Curtis distances were calculated on relative abundances, and alpha-diversity metrics (richness and Shannon) were calculated with rarefaction to account for the differences in sequencing depth in R v.4.2.2 (11) using the phyloseq v.1.38.0 (12) and vegan v.2.6-2 (13) packages.

# References

1. Greuter D, Loy A, Horn M, Rattei T. probeBase--an online resource for rRNA-targeted oligonucleotide probes and primers: new features 2016. Nucleic Acids Res. 2016;44(D1):D586-9.

2. Martin M. Cutadapt removes adapter sequences from high-throughput sequencing reads. 2011. 2011;17(1).

3. Zhang J, Kobert K, Flouri T, Stamatakis A. PEAR: a fast and accurate Illumina Paired-End reAd mergeR. Bioinformatics. 2014;30(5):614-20.

4. Schloss PD, Westcott SL, Ryabin T, Hall JR, Hartmann M, Hollister EB, et al. Introducing mothur: open-source, platform-independent, community-supported software for describing and comparing microbial communities. Appl Environ Microbiol. 2009;75(23):7537-41.

5. Rognes T, Flouri T, Nichols B, Quince C, Mahe F. VSEARCH: a versatile open source tool for metagenomics. PeerJ. 2016;4:e2584.

6. Haas BJ, Gevers D, Earl AM, Feldgarden M, Ward DV, Giannoukos G, et al. Chimeric 16S rRNA sequence formation and detection in Sanger and 454-pyrosequenced PCR amplicons. Genome Res. 2011;21(3):494-504.

7. Amir A, McDonald D, Navas-Molina JA, Kopylova E, Morton JT, Zech Xu Z, et al. Deblur Rapidly Resolves Single-Nucleotide Community Sequence Patterns. mSystems. 2017;2(2):e00191-16.

8. Wang Q, Garrity GM, Tiedje JM, Cole JR. Naive Bayesian classifier for rapid assignment of rRNA sequences into the new bacterial taxonomy. Appl Environ Microbiol. 2007;73(16):5261-7.

9. Pruesse E, Quast C, Knittel K, Fuchs BM, Ludwig W, Peplies J, et al. SILVA: a comprehensive online resource for quality checked and aligned ribosomal RNA sequence data compatible with ARB. Nucleic Acids Res. 2007;35(21):7188-96.

10. Eren AM, Morrison HG, Lescault PJ, Reveillaud J, Vineis JH, Sogin ML. Minimum entropy decomposition: unsupervised oligotyping for sensitive partitioning of high-throughput marker gene sequences. ISME J. 2015;9(4):968-79.

11. R-Core-Team. R: A Language and Environment for Statistical Computing. Vienna, Austria: R Foundation for Statistical Computing; 2023.

12. McMurdie PJ, Holmes S. phyloseq: An R Package for Reproducible Interactive Analysis and Graphics of Microbiome Census Data. PLOS ONE. 2013;8(4):e61217.

13. Oksanen J, Guillaume Blanchet F, Friendly M, Kindt R, Legendre P, McGlinn D, et al. vegan: Community Ecology Package. R package version 2.5-7 ed2020.
