# Supplementary material for: Restoration of gut microbiota with a specific synbiotic-containing infant formula in healthy Chinese infants born by cesarean section
Source: Eur J Clin Nutr. 2025 Feb 6;79(6):567–75. doi: 10.1038/s41430-025-01571-8 (PMC12151850; doi:10.1038/s41430-025-01571-8)
Supplement: Supplementary file 2 — Supplementary Figures [file 41430_2025_1571_MOESM2_ESM.docx]

# Supplementary Figures


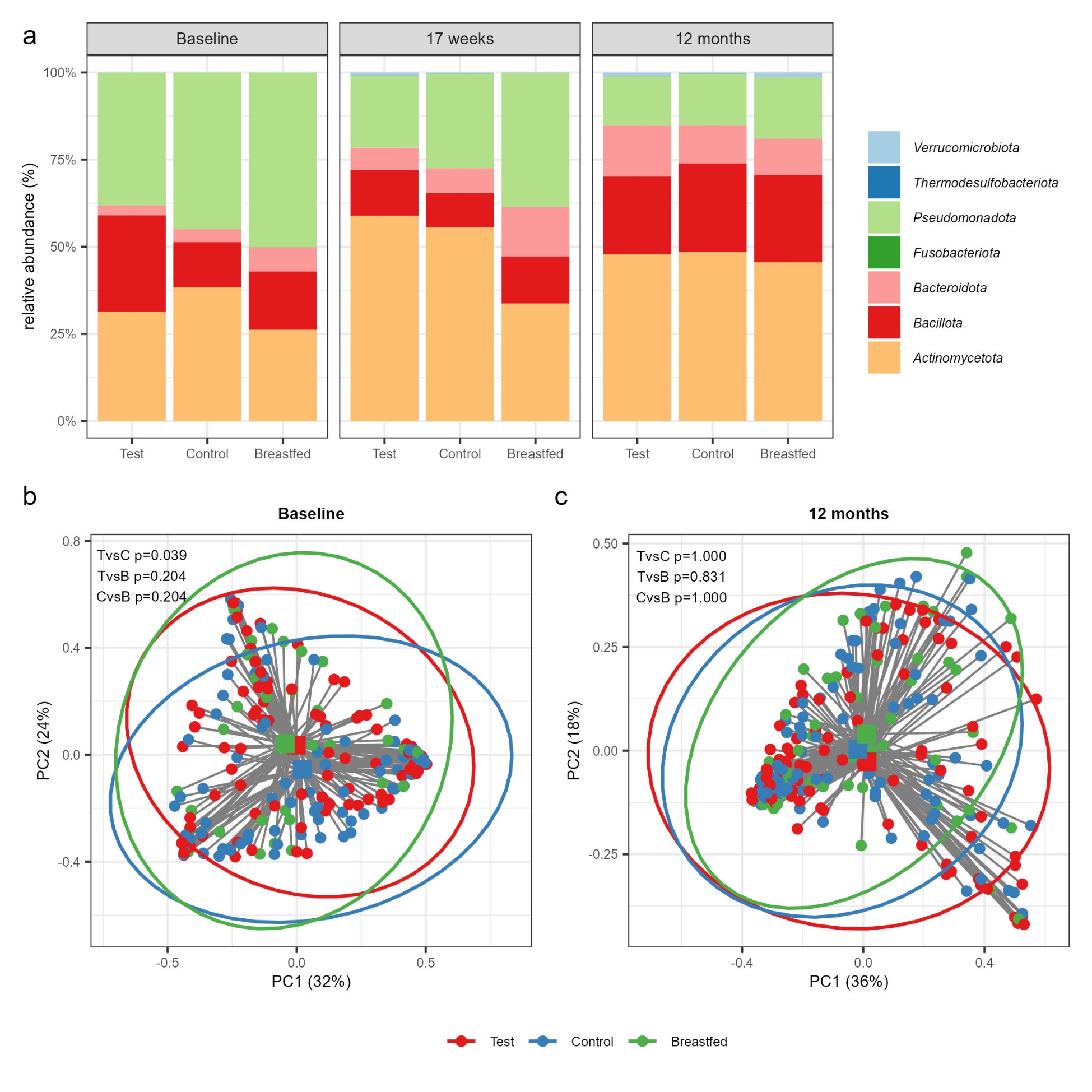


Supplementary Figure 1. Overall gut microbiota composition based on 16S rRNA-gene amplicon sequencing. (a) Stacked bar plot summarizing the average gut microbiota composition at phylum level by study group and visit. (b) Comparison of gut microbiota composition at baseline and at (b) 12 months based on a Principal Coordinate Analysis (PCoA) with Bray-Curtis sample distances calculated at genus level. The two primary axes (PC1 and PC2) together account for 56% and 54% of the total microbial variance at baseline and 12 months, respectively. Individual samples are shown as dots with their distances (grey lines) to the group centroids (squares) and the 95% confidence interval ellipses per study group. The p-values represent the results of the post-hoc comparisons from the PERMANOVA test on the group centroids with Holm’s step-down adjustment to correct for multiple testing. TvsC: Test *vs.* Control, TvsB: Test *vs.* Breastfed, CvsB: Control *vs.* Breastfed.


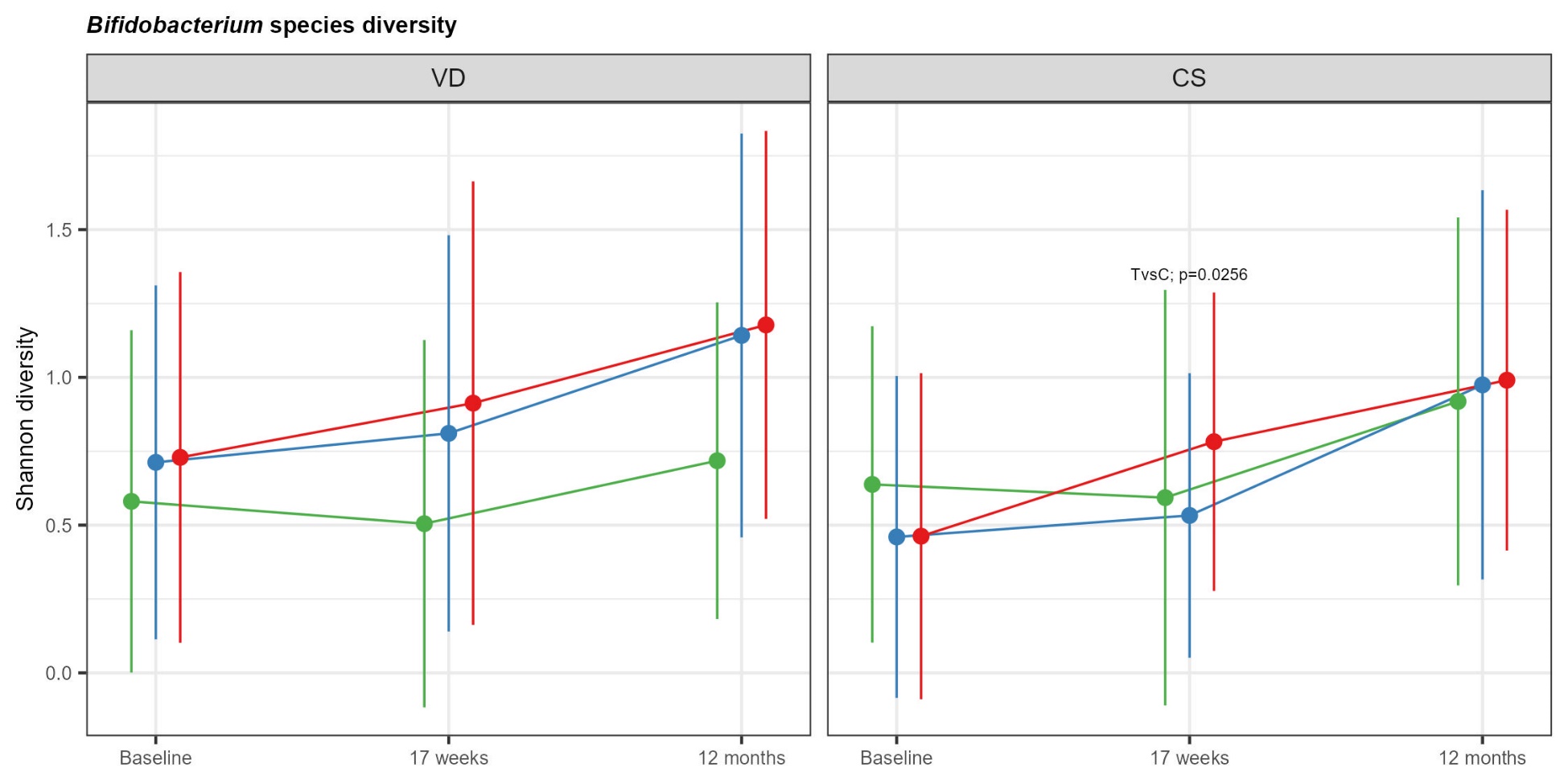


Supplementary Figure 2. Increased *Bifidobacterium* species diversity in C-section born (CS), but not vaginally-delivered (VD) infants comparing Test with Control. Line plots show bifidobacterial species diversity (mean with SD) based on the Shannon diversity index for the Test (red), Control (blue) and the Breastfed reference group (green) by birth mode with assessment of significant treatment differences (Test vs. Control, TvsC) based on a linear mixed-effects model for repeated measures.
