# Supplementary material for: Restoration of gut microbiota with a specific synbiotic-containing infant formula in healthy Chinese infants born by cesarean section
Source: Eur J Clin Nutr. 2025 Feb 6;79(6):567–75. doi: 10.1038/s41430-025-01571-8 (PMC12151850; doi:10.1038/s41430-025-01571-8)
Supplement: Supplementary file 3 — Supplementary Table 1 [file 41430_2025_1571_MOESM3_ESM.docx]

Supplementary Table 1. Number of stools with 16S rRNA gene sequencing data and sequencing depths (median and range). N = Total number of subjects in the analysis population. Nmiss is number of missing results.

| Visit | Statistics | Test  (N = 112) | Control (N = 109) | Breastfed (N = 58) |
| --- | --- | --- | --- | --- |
| Baseline | n (Nmiss) | 90 (21) | 87 (20) | 39 (19) |
|  | median (range) | 49617 (12049-65231) | 52838 (38713-63152) | 52638 (31631-65209) |
| 17 weeks | n (Nmiss) | 85 (5) | 90 (5) | 44 (8) |
|  | median (range) | 51890 (40628-62920) | 52158 (21992-64190) | 51028 (20025-65969) |
| 12 months | n (Nmiss) | 89 (0) | 92 (2) | 53 (0) |
|  | median (range) | 48359 (31123-64537) | 48361 (18511-61756) | 47079 (35277-61517) |
