# Supplementary material for: Restoration of gut microbiota with a specific synbiotic-containing infant formula in healthy Chinese infants born by cesarean section
Source: Eur J Clin Nutr. 2025 Feb 6;79(6):567–75. doi: 10.1038/s41430-025-01571-8 (PMC12151850; doi:10.1038/s41430-025-01571-8)
Supplement: Supplementary file 4 — Supplementary Table 2 [file 41430_2025_1571_MOESM4_ESM.docx]

Supplementary Table 2. Prevalence of taxa at the genus level across samples identified and assigned by 16S rRNA gene sequencing. Taxonomic assignment is given at phylum, class, order, family and genus level respectively. Number of samples (n) and percentage of samples (n%) that contained measurable amounts of the genus identified are given. The recently adopted phyla names of prokaryotes are given in the column 'phylum (ICNP)' as published by Oren, A. and G. M. Garrity (2021). Int J Syst Evol Microbiol 71(10).

| Phylum (ICNP) | phylum | class | order | family | genus | n | n% |
| --- | --- | --- | --- | --- | --- | --- | --- |
| Actinomycetota | Actinobacteriota | Actinobacteria | Bifidobacteriales | Bifidobacteriaceae | Bifidobacterium | 668 | 99.9% |
| Bacillota | Firmicutes | Bacilli | Lactobacillales | Streptococcaceae | Streptococcus | 666 | 99.6% |
| Pseudomonadota | Proteobacteria | Gammaproteobacteria | Enterobacterales | Enterobacteriaceae | Escherichia-Shigella | 661 | 98.8% |
| Bacillota | Firmicutes | Bacilli | Lactobacillales | Enterococcaceae | Enterococcus | 622 | 93.0% |
| Pseudomonadota | Proteobacteria | Gammaproteobacteria | Enterobacterales | Enterobacteriaceae | Enterobacteriaceae_unclassified | 616 | 92.1% |
| Bacillota | Firmicutes | Negativicutes | Veillonellales-Selenomonadales | Veillonellaceae | Veillonella | 602 | 90.0% |
| Pseudomonadota | Proteobacteria | Gammaproteobacteria | Enterobacterales | Enterobacteriaceae | Klebsiella | 580 | 86.7% |
| Bacillota | Firmicutes | Clostridia | Clostridiales | Clostridiaceae | Clostridium_sensu_stricto_1 | 560 | 83.7% |
| Bacteroidota | Bacteroidota | Bacteroidia | Bacteroidales | Bacteroidaceae | Bacteroides | 529 | 79.1% |
| Pseudomonadota | Proteobacteria | Gammaproteobacteria | Enterobacterales | Enterobacteriaceae | Enterobacter | 515 | 77.0% |
| Bacillota | Firmicutes | Clostridia | Lachnospirales | Lachnospiraceae | Lachnospiraceae_unclassified | 437 | 65.3% |
| Actinomycetota | Actinobacteriota | Coriobacteriia | Coriobacteriales | Eggerthellaceae | Eggerthella | 386 | 57.7% |
| Bacillota | Firmicutes | Bacilli | Lactobacillales | Lactobacillaceae | Lactobacillus | 364 | 54.4% |
| Actinomycetota | Actinobacteriota | Actinobacteria | Actinomycetales | Actinomycetaceae | Actinomyces | 347 | 51.9% |
| Actinomycetota | Actinobacteriota | Coriobacteriia | Coriobacteriales | Coriobacteriaceae | Collinsella | 347 | 51.9% |
| Bacillota | Firmicutes | Clostridia | Lachnospirales | Lachnospiraceae | Blautia | 337 | 50.4% |
| Bacillota | Firmicutes | Bacilli | Staphylococcales | Staphylococcaceae | Staphylococcus | 333 | 49.8% |
| Pseudomonadota | Proteobacteria | Gammaproteobacteria | Enterobacterales | Enterobacteriaceae | Citrobacter | 328 | 49.0% |
| Pseudomonadota | Proteobacteria | Gammaproteobacteria | Enterobacterales | Enterobacterales_unclassified | Enterobacterales_unclassified | 309 | 46.2% |
| Bacillota | Firmicutes | Clostridia | Lachnospirales | Lachnospiraceae | Lachnoclostridium | 302 | 45.1% |
| Bacillota | Firmicutes | Bacilli | Erysipelotrichales | Erysipelatoclostridiaceae | Erysipelatoclostridium | 299 | 44.7% |
| Bacillota | Firmicutes | Clostridia | Peptostreptococcales-Tissierellales | Peptostreptococcaceae | Intestinibacter | 287 | 42.9% |
| Pseudomonadota | Proteobacteria | Gammaproteobacteria | Pasteurellales | Pasteurellaceae | Haemophilus | 281 | 42.0% |
| Bacillota | Firmicutes | Clostridia | Oscillospirales | Oscillospiraceae | Flavonifractor | 278 | 41.6% |
| Bacillota | Firmicutes | Bacilli | Erysipelotrichales | Erysipelotrichaceae | Erysipelotrichaceae_ge | 260 | 38.9% |
| Bacillota | Firmicutes | Clostridia | Oscillospirales | Ruminococcaceae | Faecalibacterium | 231 | 34.5% |
| Bacillota | Firmicutes | Clostridia | Lachnospirales | Lachnospiraceae | Hungatella | 223 | 33.3% |
| Bacillota | Firmicutes | Clostridia | Peptostreptococcales-Tissierellales | Peptostreptococcaceae | Clostridioides | 220 | 32.9% |
| Bacillota | Firmicutes | Clostridia | Lachnospirales | Lachnospiraceae | Anaerostipes | 213 | 31.8% |
| Actinomycetota | Actinobacteriota | Actinobacteria | Micrococcales | Micrococcaceae | Rothia | 202 | 30.2% |
| Bacillota | Firmicutes | Clostridia | Peptostreptococcales-Tissierellales | Peptostreptococcaceae | Romboutsia | 201 | 30.0% |
| Bacillota | Firmicutes | Clostridia | Lachnospirales | Lachnospiraceae | Lachnospiraceae_ge | 199 | 29.7% |
| Bacteroidota | Bacteroidota | Bacteroidia | Bacteroidales | Tannerellaceae | Parabacteroides | 196 | 29.3% |
| Bacillota | Firmicutes | Clostridia | Clostridiales | Clostridiaceae | Clostridiaceae_unclassified | 191 | 28.6% |
| Bacillota | Firmicutes | Negativicutes | Veillonellales-Selenomonadales | Veillonellaceae | Veillonellaceae_unclassified | 170 | 25.4% |
| Bacillota | Firmicutes | Bacilli | Lactobacillales | Lactobacillales_unclassified | Lactobacillales_unclassified | 161 | 24.1% |
| Bacillota | Firmicutes | Clostridia | Lachnospirales | Lachnospiraceae | Fusicatenibacter | 159 | 23.8% |
| Bacillota | Firmicutes | Clostridia | Oscillospirales | Ruminococcaceae | Incertae_Sedis | 159 | 23.8% |
| Actinomycetota | Actinobacteriota | Actinobacteria | Propionibacteriales | Propionibacteriaceae | Cutibacterium | 124 | 18.5% |
| Bacillota | Firmicutes | Negativicutes | Veillonellales-Selenomonadales | Selenomonadaceae | Megamonas | 122 | 18.2% |
| Bacillota | Firmicutes | Clostridia | Oscillospirales | Ruminococcaceae | UBA1819 | 117 | 17.5% |
| Bacillota | Firmicutes | Negativicutes | Veillonellales-Selenomonadales | Veillonellaceae | Megasphaera | 115 | 17.2% |
| Bacillota | Firmicutes | Clostridia | Lachnospirales | Lachnospiraceae | Lachnospira | 110 | 16.4% |
| Bacillota | Firmicutes | Negativicutes | Veillonellales-Selenomonadales | Veillonellaceae | Negativicoccus | 98 | 14.6% |
| Verrucomicrobiota | Verrucomicrobiota | Verrucomicrobiae | Verrucomicrobiales | Akkermansiaceae | Akkermansia | 98 | 14.6% |
| Bacillota | Firmicutes | Clostridia | Lachnospirales | Lachnospiraceae | Agathobacter | 95 | 14.2% |
| Bacillota | Firmicutes | Clostridia | Oscillospirales | Butyricicoccaceae | Butyricicoccus | 89 | 13.3% |
| Actinomycetota | Actinobacteriota | Coriobacteriia | Coriobacteriales | Eggerthellaceae | Gordonibacter | 87 | 13.0% |
| Thermodesulfobacteriota | Desulfobacterota | Desulfovibrionia | Desulfovibrionales | Desulfovibrionaceae | Bilophila | 87 | 13.0% |
| Pseudomonadota | Proteobacteria | Gammaproteobacteria | Pseudomonadales | Moraxellaceae | Acinetobacter | 85 | 12.7% |
| Bacillota | Firmicutes | Clostridia | Oscillospirales | Ruminococcaceae | Anaerotruncus | 83 | 12.4% |
| Actinomycetota | Actinobacteriota | Actinobacteria | Actinomycetales | Actinomycetaceae | Varibaculum | 81 | 12.1% |
| Bacillota | Firmicutes | Negativicutes | Acidaminococcales | Acidaminococcaceae | Phascolarctobacterium | 80 | 12.0% |
| Pseudomonadota | Proteobacteria | Gammaproteobacteria | Burkholderiales | Sutterellaceae | Sutterella | 80 | 12.0% |
| Bacteroidota | Bacteroidota | Bacteroidia | Bacteroidales | Prevotellaceae | Prevotella | 79 | 11.8% |
| Pseudomonadota | Proteobacteria | Gammaproteobacteria | Enterobacterales | Morganellaceae | Morganella | 79 | 11.8% |
| Pseudomonadota | Proteobacteria | Gammaproteobacteria | Enterobacterales | Morganellaceae | Proteus | 79 | 11.8% |
| Bacillota | Firmicutes | Bacilli | Lactobacillales | Streptococcaceae | Lactococcus | 77 | 11.5% |
| Bacillota | Firmicutes | Clostridia | Lachnospirales | Lachnospiraceae | Roseburia | 77 | 11.5% |
| Bacillota | Firmicutes | Clostridia | Oscillospirales | Ruminococcaceae | Ruminococcus | 72 | 10.8% |
| Fusobacteriota | Fusobacteriota | Fusobacteriia | Fusobacteriales | Fusobacteriaceae | Fusobacterium | 72 | 10.8% |
| Pseudomonadota | Proteobacteria | Gammaproteobacteria | Enterobacterales | Enterobacteriaceae | Raoultella | 72 | 10.8% |
| Bacillota | Firmicutes | Clostridia | Monoglobales | Monoglobaceae | Monoglobus | 70 | 10.5% |
| Bacillota | Firmicutes | Clostridia | Oscillospirales | Ruminococcaceae | Subdoligranulum | 69 | 10.3% |
| Bacillota | Firmicutes | Negativicutes | Veillonellales-Selenomonadales | Veillonellaceae | Dialister | 69 | 10.3% |
| Bacillota | Firmicutes | Bacilli | Erysipelotrichales | Erysipelatoclostridiaceae | Coprobacillus | 65 | 9.7% |
| Pseudomonadota | Proteobacteria | Gammaproteobacteria | Burkholderiales | Sutterellaceae | Parasutterella | 60 | 9.0% |
| Pseudomonadota | Proteobacteria | Gammaproteobacteria | Enterobacterales | Enterobacteriaceae | Cronobacter | 51 | 7.6% |
| Bacteroidota | Bacteroidota | Bacteroidia | Bacteroidales | Rikenellaceae | Alistipes | 47 | 7.0% |
| Bacillota | Firmicutes | Clostridia | Lachnospirales | Lachnospiraceae | Dorea | 47 | 7.0% |
| Bacteroidota | Bacteroidota | Bacteroidia | Bacteroidales | Dysgonomonadaceae | Dysgonomonas | 42 | 6.3% |
| Bacillota | Firmicutes | Clostridia | Eubacteriales | Eubacteriaceae | Eubacterium | 42 | 6.3% |
| Bacillota | Firmicutes | Negativicutes | Acidaminococcales | Acidaminococcaceae | Acidaminococcus | 41 | 6.1% |
| Bacteroidota | Bacteroidota | Bacteroidia | Bacteroidales | Prevotellaceae | Prevotellaceae_unclassified | 40 | 6.0% |
| Bacillota | Firmicutes | Clostridia | Oscillospirales | Oscillospiraceae | Oscillibacter | 38 | 5.7% |
| Bacillota | Firmicutes | Clostridia | Lachnospirales | Lachnospiraceae | Epulopiscium | 36 | 5.4% |
| Bacillota | Firmicutes | Clostridia | Lachnospirales | Lachnospiraceae | Lachnospiraceae_ND3007_group | 35 | 5.2% |
| Bacillota | Firmicutes | Clostridia | Oscillospirales | Oscillospiraceae | Colidextribacter | 35 | 5.2% |
| Pseudomonadota | Proteobacteria | Gammaproteobacteria | Enterobacterales | Enterobacteriaceae | Enterobacteriaceae_ge | 32 | 4.8% |
| Actinomycetota | Actinobacteriota | Coriobacteriia | Coriobacteriales | Atopobiaceae | Olsenella | 28 | 4.2% |
| Pseudomonadota | Proteobacteria | Gammaproteobacteria | Pasteurellales | Pasteurellaceae | Actinobacillus | 27 | 4.0% |
| Bacillota | Firmicutes | Clostridia | Lachnospirales | Lachnospiraceae | Lachnospiraceae_UCG-004 | 26 | 3.9% |
| Thermodesulfobacteriota | Desulfobacterota | Desulfovibrionia | Desulfovibrionales | Desulfovibrionaceae | Desulfovibrio | 24 | 3.6% |
| Bacillota | Firmicutes | Clostridia | Lachnospirales | Lachnospiraceae | Sellimonas | 22 | 3.3% |
| Bacillota | Firmicutes | Bacilli | Erysipelotrichales | Erysipelatoclostridiaceae | Erysipelotrichaceae_UCG-003 | 21 | 3.1% |
| Bacillota | Firmicutes | Clostridia | Oscillospirales | Oscillospiraceae | uncultured | 20 | 3.0% |
| Bacillota | Firmicutes | Clostridia | Peptostreptococcales-Tissierellales | Peptostreptococcaceae | Paraclostridium | 20 | 3.0% |
| Bacillota | Firmicutes | Clostridia | Oscillospirales | Oscillospirales_fa | Oscillospirales_ge | 19 | 2.8% |
| Bacillota | Firmicutes | Clostridia | Lachnospirales | Lachnospiraceae | Eisenbergiella | 17 | 2.5% |
| Bacillota | Firmicutes | Clostridia | Lachnospirales | Lachnospiraceae | Tuzzerella | 17 | 2.5% |
| Bacteroidota | Bacteroidota | Bacteroidia | Bacteroidales | Prevotellaceae | Paraprevotella | 16 | 2.4% |
| Bacillota | Firmicutes | Clostridia | Clostridiales | Clostridiaceae | Clostridium_sensu_stricto_18 | 16 | 2.4% |
| Bacillota | Firmicutes | Negativicutes | Veillonellales-Selenomonadales | Veillonellaceae | Allisonella | 16 | 2.4% |
| Actinomycetota | Actinobacteriota | Coriobacteriia | Coriobacteriales | Eggerthellaceae | Senegalimassilia | 13 | 1.9% |
| Bacteroidota | Bacteroidota | Bacteroidia | Bacteroidales | Marinifilaceae | Butyricimonas | 13 | 1.9% |
| Bacteroidota | Bacteroidota | Bacteroidia | Bacteroidales | Tannerellaceae | Tannerellaceae_ge | 13 | 1.9% |
| Bacillota | Firmicutes | Clostridia | Lachnospirales | Lachnospiraceae | CAG-56 | 12 | 1.8% |
| Bacillota | Firmicutes | Clostridia | Lachnospirales | Lachnospiraceae | Coprococcus | 11 | 1.6% |
| Actinomycetota | Actinobacteriota | Coriobacteriia | Coriobacteriales | Eggerthellaceae | Slackia | 10 | 1.5% |
| Actinomycetota | Actinobacteriota | Coriobacteriia | Coriobacteriales | Coriobacteriaceae | Enorma | 7 | 1.0% |
