# Supplementary material for: Restoration of gut microbiota with a specific synbiotic-containing infant formula in healthy Chinese infants born by cesarean section
Source: Eur J Clin Nutr. 2025 Feb 6;79(6):567–75. doi: 10.1038/s41430-025-01571-8 (PMC12151850; doi:10.1038/s41430-025-01571-8)
Supplement: Supplementary file 5 — Supplementary Table 3 [file 41430_2025_1571_MOESM5_ESM.docx]

Supplementary Table 3. Prevalence and number of bifidobacterial oligotypes identified when aggregated to their species assignment. Taxonomic assignment is given at phylum, class, order, family, genus and species level respectively. Number of oligotypes assigned to the respective species level (#oligotypes), the number of samples (n) and percentage of samples (n%) that contained measurable amounts of the species identified are given. The recently adopted phyla names of prokaryotes are given in the column 'phylum (ICNP)' as published by Oren, A. and G. M. Garrity (2021). Int J Syst Evol Microbiol 71(10).

| **Phylum (ICNP)** | **phylum** | **class** | **order** | **family** | **genus** | **species** | **#oligotypes** | **n** | **n%** |
| --- | --- | --- | --- | --- | --- | --- | --- | --- | --- |
| Actinomycetota | Actinobacteria | Actinobacteria | Bifidobacteriales | Bifidobacteriaceae | Bifidobacterium | Bifidobacterium longum | 9 | 647 | 97% |
| Actinomycetota | Actinobacteria | Actinobacteria | Bifidobacteriales | Bifidobacteriaceae | Bifidobacterium | Bifidobacterium unidentified | 33 | 647 | 97% |
| Actinomycetota | Actinobacteria | Actinobacteria | Bifidobacteriales | Bifidobacteriaceae | Bifidobacterium | Bifidobacterium breve | 7 | 544 | 81% |
| Actinomycetota | Actinobacteria | Actinobacteria | Bifidobacteriales | Bifidobacteriaceae | Bifidobacterium | Bifidobacterium uncultured bacterium | 7 | 450 | 67% |
| Actinomycetota | Actinobacteria | Actinobacteria | Bifidobacteriales | Bifidobacteriaceae | Bifidobacterium | Bifidobacterium bifidum | 1 | 360 | 54% |
| Actinomycetota | Actinobacteria | Actinobacteria | Bifidobacteriales | Bifidobacteriaceae | Bifidobacterium | Bifidobacterium animalis | 1 | 211 | 32% |
| Actinomycetota | Actinobacteria | Actinobacteria | Bifidobacteriales | Bifidobacteriaceae | Bifidobacterium | Bifidobacterium dentium | 1 | 148 | 22% |
