# Supplementary material for: Restoration of gut microbiota with a specific synbiotic-containing infant formula in healthy Chinese infants born by cesarean section
Source: Eur J Clin Nutr. 2025 Feb 6;79(6):567–75. doi: 10.1038/s41430-025-01571-8 (PMC12151850; doi:10.1038/s41430-025-01571-8)
Supplement: Supplementary file 6 — Supplementary Table 4 [file 41430_2025_1571_MOESM6_ESM.docx]

Supplementary Table 4. Stool physiology data (pH, short chain fatty acids, lactic acids and secretory IgA) measured in stools of the analysis population. Descriptive statistics are shown for the different parameters and the p-values based on the comparisons of Test with Control (TvsC) at the different visits. Mean with standard deviation (SD) are shown for the parameters that were compared using a linear mixed-effects model for repeated measures (MMRM), while median plus the interquartile ranges (Q1; Q3) are shown for parameters that could not be analyzed adequately with a MMRM and were compared by a Wilcoxon Rank Sum test instead. All parameters except for pH were log-transformed prior to running the MMRM. P-values < 0.05 are shown in bold. N = Number of subjects of the analysis population. Nmiss is number of missing results.

| Parameter | Visit | Statistics | Test (N = 112) | Control (N = 109) | P-value TvsC | Breastfed (N = 58) |
| --- | --- | --- | --- | --- | --- | --- |
| pH | Baseline | n (Nmiss) | 104 (7) | 104 (3) |  | 50 (8) |
|  |  | Mean (SD) | 6.13 (0.84) | 6.08 (0.77) | 0.6764 | 6.15 (0.85) |
|  | 17 weeks | n (Nmiss) | 89 (1) | 93 (2) |  | 47 (5) |
|  |  | Mean (SD) | 6.19 (0.89) | 6.18 (0.90) | 0.9264 | 6.04 (0.93) |
|  | 12 months | n (Nmiss) | 89 (0) | 94 (0) |  | 53 (0) |
|  |  | Mean (SD) | 6.62 (0.92) | 6.59 (0.95) | 0.8369 | 6.38 (0.90) |
| secretory IgA (µg/g) | Baseline | n (Nmiss) | 89 (22) | 86 (21) |  | 39 (19) |
|  |  | Median (Q1; Q3) | 449 (1; 2273) | 518 (1; 1750) | 0.644 | 2732 (1689; 4939) |
|  | 17 weeks | n (Nmiss) | 79 (11) | 87 (8) |  | 43 (9) |
|  |  | Median (Q1; Q3) | 911 (359; 1508) | 880 (601; 1471) | 0.319 | 2609 (888; 4653) |
|  | 12 months | n (Nmiss) | 88 (1) | 93 (1) |  | 50 (3) |
|  |  | Median (Q1; Q3) | 317 (119; 669) | 331 (206; 703) | 0.259 | 538 (259; 1369) |
| D-lactate (mmol/kg) | Baseline | n (Nmiss) | 83 (28) | 82 (25) |  | 37 (21) |
|  |  | Median (Q1; Q3) | 1.7 (0.8; 4.9) | 2.3 (0.8; 6.4) | 0.279 | 0.7 (0.4; 1.2) |
|  | 17 weeks | n (Nmiss) | 78 (12) | 86 (9) |  | 43 (9) |
|  |  | Median (Q1; Q3) | 3.0 (1.0; 5.9) | 3.8 (1.9; 9.1) | 0.063 | 1.3 (0.6; 2.8) |
|  | 12 months | n (Nmiss) | 87 (2) | 91 (3) |  | 50 (3) |
|  |  | Median (Q1; Q3) | 0.8 (0.2; 1.9) | 1.0 (0.4; 4.4) | 0.165 | 1.5 (0.7; 4.7) |
| L-lactate (mmol/kg) | Baseline | n (Nmiss) | 83 (28) | 82 (25) |  | 37 (21) |
|  |  | Mean (SD) | 9.81 (10.81) | 11.93 (11.74) | 0.8297 | 5.89 (9.53) |
|  | 17 weeks | n (Nmiss) | 78 (12) | 86 (9) |  | 43 (9) |
|  |  | Mean (SD) | 11.22 (9.09) | 16.74 (10.45) | **0.0088** | 12.33 (10.89) |
|  | 12 months | n (Nmiss) | 87 (2) | 91 (3) |  | 50 (3) |
|  |  | Mean (SD) | 4.24 (7.42) | 6.30 (9.60) | 0.1202 | 5.98 (8.20) |
| Acetic Acid (mmol/kg) | Baseline | n (Nmiss) | 98 (13) | 100 (7) |  | 44 (14) |
|  |  | Mean (SD) | 43.09 (30.70) | 53.96 (38.35) | 0.4388 | 26.43 (21.61) |
|  | 17 weeks | n (Nmiss) | 83 (7) | 93 (2) |  | 46 (6) |
|  |  | Mean (SD) | 72.60 (35.64) | 89.53 (54.39) | **0.0067** | 39.61 (24.32) |
|  | 12 months | n (Nmiss) | 88 (1) | 94 (0) |  | 53 (0) |
|  |  | Mean (SD) | 94.71 (52.01) | 93.80 (41.97) | 0.4238 | 84.05 (35.18) |
| Butyric Acid (mmol/kg) | Baseline | n (Nmiss) | 98 (13) | 100 (7) |  | 44 (14) |
|  |  | Mean (SD) | 3.29 (5.39) | 3.02 (6.81) | 0.4867 | 2.02 (7.00) |
|  | 17 weeks | n (Nmiss) | 83 (7) | 93 (2) |  | 46 (6) |
|  |  | Mean (SD) | 3.47 (4.97) | 2.95 (3.89) | 0.2462 | 1.35 (1.88) |
|  | 12 months | n (Nmiss) | 88 (1) | 94 (0) |  | 53 (0) |
|  |  | Mean (SD) | 11.05 (10.73) | 8.70 (7.57) | 0.198 | 8.42 (7.71) |
| Propionic Acid (mmol/kg) | Baseline | n (Nmiss) | 98 (13) | 100 (7) |  | 44 (14) |
|  |  | Mean (SD) | 9.71 (10.26) | 7.83 (8.55) | 0.2384 | 3.71 (3.26) |
|  | 17 weeks | n (Nmiss) | 83 (7) | 93 (2) |  | 46 (6) |
|  |  | Mean (SD) | 12.21 (8.17) | 14.23 (12.25) | 0.5149 | 3.46 (4.00) |
|  | 12 months | n (Nmiss) | 88 (1) | 94 (0) |  | 53 (0) |
|  |  | Mean (SD) | 18.71 (12.69) | 17.95 (13.32) | 0.3979 | 15.02 (9.72) |
| Valeric Acid (mmol/kg) | Baseline | n (Nmiss) | 98 (13) | 100 (7) |  | 44 (14) |
|  |  | Median (Q1; Q3) | 0.24 (0.22; 0.27) | 0.24 (0.22; 0.26) | 0.39 | 0.23 (0.22; 0.25) |
|  | 17 weeks | n (Nmiss) | 83 (7) | 93 (2) |  | 46 (6) |
|  |  | Median (Q1; Q3) | 0.25 (0.23; 0.28) | 0.24 (0.22; 0.27) | 0.179 | 0.22 (0.21; 0.25) |
|  | 12 months | n (Nmiss) | 88 (1) | 94 (0) |  | 53 (0) |
|  |  | Median (Q1; Q3) | 0.42 (0.28; 0.94) | 0.37 (0.27; 0.68) | 0.493 | 0.32 (0.25; 0.74) |
| Isobutyric Acid (mmol/kg) | Baseline | n (Nmiss) | 98 (13) | 100 (7) |  | 44 (14) |
|  |  | Mean (SD) | 0.312 (0.528) | 0.256 (0.478) | 0.3101 | 0.075 (0.049) |
|  | 17 weeks | n (Nmiss) | 83 (7) | 93 (2) |  | 46 (6) |
|  |  | Mean (SD) | 0.585 (0.620) | 0.498 (0.880) | **0.0154** | 0.141 (0.226) |
|  | 12 months | n (Nmiss) | 88 (1) | 94 (0) |  | 53 (0) |
|  |  | Mean (SD) | 1.367 (1.075) | 1.172 (1.013) | 0.1952 | 1.196 (1.217) |
| Isovaleric Acid (mmol/kg) | Baseline | n (Nmiss) | 98 (13) | 100 (7) |  | 44 (14) |
|  |  | Mean (SD) | 0.195 (0.361) | 0.198 (0.498) | 0.2874 | 0.030 (0.031) |
|  | 17 weeks | n (Nmiss) | 83 (7) | 93 (2) |  | 46 (6) |
|  |  | Mean (SD) | 0.575 (0.799) | 0.352 (0.698) | **0.0006** | 0.086 (0.203) |
|  | 12 months | n (Nmiss) | 88 (1) | 94 (0) |  | 53 (0) |
|  |  | Mean (SD) | 1.015 (0.936) | 0.883 (0.922) | 0.1264 | 1.017 (1.097) |
